# High-Sensitivity C-Reactive Protein and its Association with Diabetic Retinopathy: A Cross-sectional Study

**DOI:** 10.34172/aim.34506

**Published:** 2025-08-01

**Authors:** Zahra Faghih Abdollahi, Mansour Babaei, Neda Meftah, Hoda Shirafkan, Seyed Ahmad Rasoulinejad

**Affiliations:** ^1^Student Research Committee, Babol University of Medical Sciences, Babol, Iran; ^2^Department of Internal Medicine, Ayatollah Rouhani Hospital, Division of Rheumatology, Babol University of Medical Sciences, Babol, Iran; ^3^Department of Internal Medicine, School of Medicine, Babol University of Medical Sciences, Babol, Iran; ^4^Social Determinants of Health Research Center, Health Research Institute, Babol University of Medical Sciences, Babol, Iran; ^5^Department of Ophthalmology, Ayatollah Rouhani Hospital, Babol University of Medical Sciences, Babol, Iran

**Keywords:** Diabetic retinopathy, High-sensitivity C-reactive protein, Inflammation, Inflammatory biomarkers, Type 2 diabetes mellitus

## Abstract

**Background:**

Chronic low-grade inflammation is implicated in diabetic microvascular complications, but the relationship between circulating high-sensitivity C-reactive protein (hs-CRP) and diabetic retinopathy (DR) in type 2 diabetes mellitus (T2DM) remains incompletely defined. We evaluated whether plasma hs-CRP levels are independently associated with DR presence and severity in a cohort of T2DM patients.

**Methods:**

In this analytical cross-sectional study, 149 T2DM patients referring to endocrinology and ophthalmology clinics at Babol University of Medical Sciences (April 2022–June 2023) were categorized into no DR (n=50), non-proliferative DR (NPDR; n=49), and proliferative DR (PDR; n=50) groups. Comprehensive ophthalmic examination classified DR stage. We measured hs-CRP (low: 0-1 mg/L, moderate: 1-3 mg/mL, high:>3 mg/mL), HbA1c, fasting blood sugar (FBS), and total cholesterol in fasting blood samples. Statistical analysis was performed with SPSS v.22.

**Results:**

Mean hs-CRP concentrations rose progressively with DR severity: 2.71±1.14 mg/L (no DR), 4.89±5.31 mg/L (NPDR), and 10.60±9.24 mg/L (PDR; *P*=0.023). After adjusting for age, sex, diabetes duration, HbA1c, BMI, hypertension, smoking, cholesterol, and treatment, each 1 mg/L increase in hs-CRP was associated with 1.40-fold higher odds of DR (OR 1.40; 95% CI 1.08–1.94; *P*=0.011). Other independent predictors included longer diabetes duration (OR 1.19 per year; 95% CI 1.10–1.29; *P*<0.001), higher HbA1c (OR 1.62 per %; 95% CI 1.06–2.48; *P*=0.023), and female sex (OR 3.25; 95% CI 1.11–9.52; *P*=0.031).

**Conclusion:**

High hs-CRP levels correlate with DR severity in T2DM, highlighting inflammation’s role and potential for early detection strategies.

## Introduction

 Diabetic retinopathy (DR) is one of the leading causes of vision impairment worldwide and the primary cause of visual loss among diabetic individuals aged 25 years and older.^1^ Vision loss resulting from DR may be secondary to macular edema (ME), hemorrhage from neovascularization, retinal detachment, or neovascular glaucoma. The vast majority of patients with DR remain asymptomatic until the disease has significantly progressed.^2^ Given the potential for rapid progression and the effectiveness of early intervention in slowing or reversing vision loss, regular screening for retinal complications in diabetic patients is crucial. The rising regional burden of diabetes in Asia reinforces the need for earlier identification and management of its microvascular complications, including DR.^3^ Approximately 93 million individuals worldwide are affected by DR. The prevalence of DR is about 77.3% in patients with type 1 diabetes and 25.1% in those with type 2 diabetes mellitus (T2DM).^4^ Various ocular disorders, including immune-related uveitis from checkpoint inhibitors, demonstrate the crucial impact of inflammation in ocular pathology.^5^

 DR is categorized into two major forms: non-proliferative and proliferative, based on the presence or absence of abnormal neovascularization originating from the retina. Non-proliferative DR (NPDR) is characterized by microaneurysms, retinal hemorrhages, hard exudates, cotton wool spots, and intraretinal microvascular abnormalities. Visual impairment in NPDR primarily results from ME. NPDR is further classified into mild, moderate, severe, and very severe stages based on the risk of progression to proliferative DR (PDR).^6^ In contrast, PDR is defined by the presence of neovascularization, which may lead to preretinal or vitreous hemorrhage, fibrotic changes, and tractional retinal detachment, all of which can cause severe and irreversible vision loss.^7^

 ME may occur at any stage of DR and is defined by retinal thickening and edema involving the macula. It can be detected using fundus examination, fluorescein angiography, and optical coherence tomography (OCT).^8^ Risk factors for DR include duration of diabetes, poor glycemic control (measured by HbA1c), hypertension, dyslipidemia, obesity, smoking, and genetic predisposition.^9^ Chronic hyperglycemia leads to the activation of alternative metabolic pathways such as the polyol pathway, oxidative stress, protein kinase C activation, and non-enzymatic glycation, which result in endothelial dysfunction and microvascular damage. These processes ultimately increase vascular permeability, capillary occlusion, and retinal ischemia, contributing to the development and progression of DR.^10^

 Among inflammatory biomarkers, high-sensitivity C-reactive protein (hs-CRP) has gained attention. CRP is an acute-phase protein synthesized by the liver in response to interleukin-6 and other pro-inflammatory cytokines.^10^ Elevated hs-CRP levels have been associated with systemic inflammation, cardiovascular disease, and, more recently, microvascular complications of diabetes, including diabetic neuropathy, nephropathy, and retinopathy. While several studies suggest a positive association between hs-CRP levels and the incidence or severity of DR, others have reported inconsistent or even inverse relationships, highlighting the need for further investigation.^11-13^ This study aims to evaluate the association between hs-CRP plasma levels and DR in patients with T2DM to gain a clearer understanding of the role of inflammation and endothelial damage in the pathogenesis of DR. These contrasting results may arise because of ethnic and genetic differences in baseline CRP expression, differences in analytic methods, and variations in the assessment of DR. The aim of this study was to assess whether plasma hs-CRP levels were independently associated with the presence and severity of DR in T2DM patients, adjusted for possible confounders. This would help to clarify the role of systemic inflammation in the DR pathophysiology and also guide to understand the conflicting evidence reported in the literature.

## Materials and Methods

###  Study Design and Setting

 This analytical cross-sectional study was performed in Babol, northern Iran, during the period from April 2022 to June 2023. The target population had a previous diagnosis of T2DM and referred to either the endocrine clinic or any of the ophthalmology associates/private offices, which are all associated with the Babol University of Medical Sciences. The inclusion criteria included a diagnosis of T2DM, and the individuals agreed to participate and signed the informed consent form. The exclusion criteria were being diagnosed with type 1 diabetes, currently pregnant or lactating, having a recent diagnosis of an infection, malignancy, acute or chronic inflammation state, hepatic or renal failure, and using medications affecting hs-CRP levels, including corticosteroids. To facilitate the assessment in the totality of the DR spectrum, patients who did not have retinopathy were included as a reference group. These patients tended to be referred for a screening appointment or management for diabetes-related symptoms.

###  Sample Size Calculation 

 The sample size was estimated based on previously published data by Nada andAbdel-Moety,^14^ that examined differences in hs-CRP levels across DR stages in patients with T2DM. The sample size was calculated based on this formula: n = 2(k−1)(Z_1−α/2​_ + Z_1−β_​)^2^/kf^2^, where k equals 3, representing the number of groups; Z_1−α/2​_ is 1.96, the critical value corresponding to a 95% confidence level; Z_1-β_ is 0.91, the critical value for 70% statistical power; and f is 0.52, indicating a large effect size according to Cohen’s conventions (n = 41 participants per group). To enhance statistical robustness and account for potential confounders and inter-individual variability in hs-CRP, we aimed to recruit approximately 150 participants, ultimately including 149 patients distributed nearly equally across the three DR groups.

###  Sampling Method, Data Collection, and Ophthalmic Examination

 A total of 149 eligible patients were enrolled using a census sampling method. Participants were categorized into three groups based on the presence and severity of DR: T2DM patients without retinopathy, T2DM patients with NPDR, and patients with PDR. After obtaining informed consent, patients underwent a comprehensive ophthalmic examination. Following pharmacological pupil dilation, retinal evaluation was performed by a vitreoretinal fellowship-trained ophthalmologist using a slit-lamp biomicroscope with a 90-diopter lens. Diagnosis and classification of DR were based on the presence of microaneurysms, retinal hemorrhages, exudates, ME, and neovascularization.

###  Laboratory Assessment and Demographic Variables

 Blood samples were collected to measure the following parameters: hs-CRP, fasting blood sugar (FBS), glycated hemoglobin (HbA1c), and total cholesterol (ParsAzmoon biochemical kit, Iran). Internal quality controls were used per manufacturer protocol. Also, hs-CRP level was categorized into three groups: 0-1 mg/L as low, 1-3 mg/L as moderate, and > 3 mg/L as high levels.

 In addition to laboratory data, demographic and clinical information was recorded, including age, gender, duration of diabetes, body mass index (BMI), smoking status, blood pressure status (hypertension defined as > 140/90 mm Hg), and type of antidiabetic treatment (oral, insulin, or combined therapy).

###  Statistical Analysis

 Data were analyzed using the SPSS software version 22. Descriptive statistics were used to summarize demographic and clinical variables. Quantitative data were expressed as mean ± standard deviation and compared using Student’s t-test or ANOVA, as appropriate. Categorical data were compared using the chi-square test. Logistic regression analysis was used to assess the association between hs-CRP levels and DR while controlling for potential confounders such as age, gender, duration of diabetes, HbA1c, BMI, and hypertension. The dependent variable in the regression model was defined as a binary outcome representing the presence of any DR (yes/no). Backward elimination was then applied with an entry p-value cut-off of 0.20 and a removal p-value cut-off of 0.10.

## Results

###  Demographic Characteristics 

 A total of 149 patients with T2DM were included: 50 without DR, 49 with NPDR, and 50 with PDR. Overall, 45 patients (30.2%) were male and 104 (69.8%) were female. The mean age was 59.36 ± 10.57 years (range 36–89 years), and age increased significantly with DR severity: 54.76 ± 9.85 years in the no-DR group, 61.30 ± 10.53 years in NPDR, and 62.06 ± 9.95 years in PDR (*P* < 0.001). The mean duration of diabetes was 11.73 ± 7.49 years, rising from 6.82 ± 5.74 years (no DR) to 11.06 ± 6.60 years (NPDR) and 17.32 ± 6.06 years (PDR) (*P* < 0.001).

 Treatment modality differed significantly across the groups (*P* < 0.001). Among patients without DR, 96% were managed with oral agents alone, versus 71.4% in NPDR and 44% in PDR. Insulin monotherapy was used by 4%, 12.2%, and 34% of the no-DR, NPDR, and PDR groups, respectively, while combined oral + insulin therapy was employed by 0%, 16.3%, and 22% of those groups.

 Most participants were non-smokers (93.3% overall), with smokers comprising 4% of the no-DR group, 10.2% of NPDR, and 6% of PDR (p = 0.453). Hypertension prevalence also rose with retinopathy severity (from 42% in no-DR to 46.9% in NPDR and 60% in PDR), although this difference did not reach statistical significance (*P* = 0.178).

 BMI showed a modest but significant increase across DR categories (*P* = 0.025), with mean BMI values of 27.35 ± 4.01 kg/m^2^ in no-DR, 27.76 ± 2.34 kg/m^2^ in NPDR, and 29.24 ± 4.13 kg/m^2^ in PDR (Table 1).

**Table 1:** Baseline Demographic and Treatment Profiles Across DR Severity Groups.

**Variable**		**No DR (n=50)**	**NPDR (n=49)**	**PDR (n=50)**	***P*** ** value**
Mean Age (years)		54.76 ± 9.85	61.30 ± 10.53	62.06 ± 9.95	< 0.001^*^
Gender	Male	14 (28.0%)	19 (38.8%)	12 (24.0%)	0.255^#^
	Female	36 (72.0%)	30 (61.2%)	38 (76.0%)	
Duration of diabetes (years)		6.82 ± 5.74	11.06 ± 6.60	17.32 ± 6.06	< 0.001^*^
Treatment type	Oral only	48 (96.0%)	35 (71.4%)	22 (44.0%)	< 0.001^#^
	Insulin only	2 (4.0%)	6 (12.2%)	17 (34.0%)	
	Oral + Insulin	0 (0.0%)	8 (16.3%)	11 (22.0%)	
Smoking	Non-smoker	48 (96.0%)	44 (89.8%)	47 (94.0%)	0.453^#^
	Smoker	2 (4.0%)	5 (10.2%)	3 (6.0%)	
Hypertension	Absent	29 (58.0%)	26 (53.1%)	20 (40.0%)	0.178^#^
	Present	21 (42.0%)	23 (46.9%)	30 (60.0%)	
BMI (kg/m^2^)		27.35 ± 4.01	27.76 ± 2.34	29.24 ± 4.13	0.025^*^

^*^One-way ANOVA; ^#^Chi-square test.

###  Association of Inflammatory and Metabolic Parameters with Diabetic Retinopathy Severity

 In patients without DR, mean hs-CRP was 2.71 ± 1.14 mg/L and rose progressively to 4.89 ± 5.31 mg/L in those with NPDR and 10.60 ± 9.24 mg/L in PDR (*P* = 0.023). Glycemic control, as reflected by HbA1c, also worsened across the groups: 7.25 ± 1.29% in patients without DR versus 7.93 ± 1.29% in NPDR and 8.50 ± 1.51% in PDR groups (*P* < 0.001). FBS followed a similar upward trend from 149.20 ± 51.24 mg/L in patients without DR, to 151.10 ± 51.67 mg/L in NPDR, and 184.34 ± 76.26 mg/L in PDR groups (*P* = 0.006). By contrast, mean total cholesterol levels did not differ significantly among the three groups (182.38 ± 46.34 vs. 187.16 ± 40.60 vs. 171.16 ± 43.78 mg/L; *P* = 0.176) (Table 2).

**Table 2:** Inflammatory and Metabolic Parameters Across Diabetic Retinopathy Severity Groups

**Parameter**	**No DR (n=50)**	**NPDR (n=49)**	**PDR (n=50)**	***P*** value^*^
hs-CRP (mg/L)	2.71 ± 1.14	4.89 ± 5.31	10.60 ± 9.24	0.023
HbA1c (%)	7.25 ± 1.29	7.93 ± 1.29	8.50 ± 1.51	< 0.001
FBS (mg/dL)	149.20 ± 51.24	151.10 ± 51.67	184.34 ± 76.26	0.006
Cholesterol (mg/dL)	182.38 ± 46.34	187.16 ± 40.60	171.16 ± 43.78	0.176

^*^One-way ANOVA.

###  Association Between hs -CRP and Diabetic Retinopathy Across Subgroups

 Subgroup analysis of hs-CRP levels demonstrated that inflammation increased with the severity of DR in several patient categories. Among patients under 60 years of age, hs-CRP rose from 2.74 ± 1.24 mg/L in those without DR to 5.47 ± 4.29 mg/L in the PDR group. In patients aged 60 and above, hs-CRP reached 6.56 ± 11.66 mg/L in PDR. Despite this upward trend, age subgroup differences were not statistically significant (*P* = 0.800).

 Female patients had higher hs-CRP values than males, especially in the PDR group (7.08 ± 10.36 vs. 3.02 ± 2.51 mg/L), though this gender-based difference was not statistically significant (*P* = 0.410). Diabetes duration was associated with markedly elevated hs-CRP in patients with 6–10 years and 11–15 years of disease, with PDR values of 12.25 ± 20.79 mg/L and 8.10 ± 8.24 mg/L, respectively (*P* = 0.130).

 Among BMI categories, the highest hs-CRP was observed in obese individuals with NPDR (7.18 ± 5.31 mg/L), but differences across BMI strata were not statistically significant (*P* = 0.800). Smokers showed slightly lower hs-CRP levels than non-smokers across DR groups (e.g., 3.40 ± 2.51 vs. 6.28 ± 9.50 mg/L in PDR), with no significant difference (*P* = 0.790). Similarly, hypertension showed no significant association (*P* = 0.130), although hypertensive patients with PDR had hs-CRP levels of 7.57 ± 11.55 mg/L compared to 3.91 ± 2.87 mg/L in normotensives.

 Regarding treatment type, patients on oral medications alone had hs-CRP levels of 7.23 ± 12.17 mg/L in PDR, while those receiving both oral and insulin therapy had the highest value in NPDR (9.23 ± 9.90 mg/L). The overall difference by treatment modality showed a borderline trend (*P* = 0.100). Poor glycemic control was associated with higher hs-CRP in PDR (6.75 ± 9.67 mg/L) compared to patients with good control (1.35 ± 1.36 mg/L). Similarly, patients with cholesterol ≥ 200 mg/L had elevated hs-CRP levels in PDR (11.24 ± 17.28 mg/L), while those with cholesterol < 200 mg/L showed lower values (4.48 ± 3.63 mg/L), with a near-significant p-value of 0.060 (Table 3).

**Table 3:** Association Between hs-CRP and Diabetic Retinopathy Across Subgroups

**Subgroup**		**No DR (n=50)**	**NPDR (n=49)**	**PDR (n=50)**	***P*** value^*^
Age	< 60 years	2.74 ± 1.24	5.18 ± 4.64	5.47 ± 4.29	0.800
	≥ 60 years	2.65 ± 0.93	4.65 ± 5.87	6.56 ± 11.66	
Sex	Male	2.40 ± 1.32	3.11 ± 1.41	3.02 ± 2.51	0.410
	Female	2.83 ± 1.07	6.01 ± 6.48	7.08 ± 10.36	
Duration of diabetes	< 5 years	2.62 ± 1.07	3.75 ± 3.22	0.00 ± 0.00	0.130
	6–10 years	3.05 ± 1.47	3.76 ± 2.76	12.25 ± 20.79	
	11–15 years	2.55 ± 1.09	6.87 ± 5.69	8.10 ± 8.24	
	16–20 years	2.50	6.56 ± 8.92	3.48 ± 1.91	
	> 20 years	2.50 ± 0.70	3.66 ± 2.02	3.51 ± 2.57	
BMI category	Underweight	2.80	3.41 ± 1.55	3.54	0.800
	Normal	2.41 ± 0.72	5.07 ± 6.04	7.18 ± 2.01	
	Overweight	2.59 ± 1.27	3.54 ± 3.51	5.48 ± 12.80	
	Obese	3.25 ± 1.16	7.18 ± 5.31	6.10 ± 4.09	
Smoking	No	2.66 ± 1.51	5.06 ± 5.57	6.28 ± 9.50	0.790
	Yes	3.75 ± 0.24	3.34 ± 1.05	3.40 ± 2.51	
Hypertension	No	2.71 ± 1.24	5.46 ± 6.93	3.91 ± 2.87	0.130
	Yes	2.71 ± 1.02	4.24 ± 2.48	7.57 ± 11.55	
Treatment type	Oral only	2.70 ± 1.17	3.92 ± 3.74	7.23 ± 12.17	0.100
	Insulin only	2.90 ± 0.14	4.71 ± 1.27	5.82 ± 7.34	
	Oral + Insulin	—	9.23 ± 9.90	4.30 ± 3.90	
HbA1c control	Good	2.36 ± 1.35	4.09 ± 5.09	1.35 ± 1.36	0.300
	Poor	2.98 ± 0.88	5.12 ± 5.41	6.75 ± 9.67	
Cholesterol level	< 200 mg/dL	2.59 ± 1.12	4.04 ± 3.60	4.48 ± 3.63	0.060
	≥ 200 mg/dL	3.02 ± 1.19	6.35 ± 7.28	11.24 ± 17.28	

^*^One-way ANOVA.

 In categorizing hs-CRP into low, moderate, and high levels, a significant association was found between hs-CRP levels and DR severity (χ^2^(4) = 37.87, *P* < .001, Cramer’s V = 0.36). Patients with high hs-CRP had elevated PDR rates (46.1% proliferative), whereas moderate hs-CRP was associated with lower risk (12.7% proliferative).

###  Multivariable Logistic Regression Analysis of Risk Factors for Diabetic Retinopathy

 To identify the independent predictors of DR, a multivariable logistic regression analysis was performed using a backward elimination method. The variables initially entered into the model included age, sex, duration of diabetes, hs-CRP, HbA1c, BMI, cholesterol, smoking status, hypertension, and type of treatment. Given that hs-CRP was the primary exposure of interest, it was retained in the model regardless of statistical significance to ensure that its effect on DR could be assessed independently of the selection process.

 After stepwise elimination, five variables remained in the final model. The results showed that hs-CRP was a significant independent predictor of DR; for every 1 mg/L increase, the odds of having DR increased by 1.40 times (OR = 1.40, 95% CI: 1.08–1.94; *P* = 0.011). Similarly, female sex was associated with a 3.25-fold increase in the risk of DR (OR = 3.25, 95% CI: 1.11–9.52; *P* = 0.031).

 Each additional year of diabetes increased the odds of DR by 19% (OR = 1.19, 95% CI: 1.10–1.29; *P* < 0.001). HbA1c also showed a significant association, while each 1% increase in HbA1c raised the odds of DR by 62% (OR = 1.62, 95% CI: 1.06–2.48; *P* = 0.023). Age showed a trend toward significance (OR = 1.046, 95% CI: 0.998–1.09; *P* = 0.059), suggesting a possible modest effect on DR risk (Table 4).

**Table 4:** Multivariable Logistic Regression Model Predicting Diabetic Retinopathy.

**Factor**	**OR**	***P*** ** value**	95% (CI)^*^
hs-CRP (per mg/L)	1.40	0.011	1.08 – 1.94
Female sex	3.25	0.031	1.11 – 9.52
Age (per year)	1.046	0.059	0.998 – 1.09
Duration of diabetes (per year)	1.19	< 0.001	1.10 – 1.29
HbA1c (per %)	1.62	0.023	1.06 – 2.48

CI, confidence interval; OR, odds ratio.^*^Multivariate logistic regression (with backward elimination).

## Discussion

 Our study found a clear positive association between serum hs-CRP levels and the severity of DR. Patients with more advanced DR (severe NPDR and PDR) had significantly higher hs-CRP concentrations than those with milder or no retinopathy. This graded increase in hs-CRP with DR stage suggests that systemic inflammation intensifies as retinal disease progresses. In the Singapore Malay Eye Study, Lim et al found that higher CRP (and higher BMI) were paradoxically associated with lower DR prevalence and severity.^12^ Likewise, a cohort of Chinese patients showed an inverse CRP–DR relationship: after multivariable adjustment, the highest CRP quartile had a 25–40% reduced odds of any DR.^15^ We suggest that these surprising findings could stem from differences in ethnic background, body composition, or unmeasured confounders. Just as baseline CRP levels and inflammatory profiles may differ between ethnicities due to genetic polymorphism, we need to recognize that there is no such thing as an ethnicity-independent effect estimate. The ways we can adjust for confounding factors may differ in how we account for obesity, insulin resistance, or renal functions, which may also have the effect of changing either the direction or the strength of an effect. Similarly, the heterogeneity in the way DR was assessed (i.e. fundus photographs versus clinical grading, binary versus ordinal data classifications of DR severity) can explain the differences in how we categorize the outcomes. Indeed, an expert review cited the discordant CRP–DR associations, noting changes in BMI, glycemic control, and/or controlled study design.^16^ Future research should integrate genetic and metabolic profiling to clarify population-specific modifiers of the CRP–DR link. Importantly, our data align with the majority of reports (including the pooled analysis by Song et al^17^) that support a positive CRP–DR link. The conflicting outliers underline that CRP’s role may be modified by population characteristics and must be interpreted cautiously.

 We examined whether the CRP–DR relationship varied by age, sex, BMI, diabetes duration, treatment modality, or glycemic control, as prior studies have suggested such effects. In general, older age, longer diabetes duration, and worse glycemic indices are known risk factors for DR, and they also tend to be associated with higher CRP. In our sample, hs-CRP increased with patient age and with diabetes duration and HbA1c level, consistent with previous reports.^16,18^ However, the association between hs-CRP and DR severity remained significant even after adjusting for these factors, suggesting an independent effect. Sex differences were minimal in our cohort; CRP levels did not differ meaningfully between men and women when matched for other variables, nor did the CRP–DR trend significantly differ by gender. In the literature, CRP tends to be higher in women on average, but women do not uniformly have higher DR rates; one study speculated that higher CRP might even partly explain lower DR prevalence in women.^19^ BMI is a key confounder because adiposity drives chronic inflammation. We observed, as expected, that higher BMI was associated with higher hs-CRP. Other authors have also highlighted BMI’s complex role: Lim et al found that both BMI and CRP showed inverse associations with DR, raising the possibility that obesity-related metabolic factors modify DR risk.^12^ In contrast, Yang et al adjusted for BMI (and many other covariates) and still observed an inverse CRP–DR link.^20^ Our data cannot definitively resolve these contradictions; we found that the CRP–DR relationship holds in both lean and overweight subgroups. Lastly, treatment modality (insulin vs. oral agents) and other comorbidities (hypertension, dyslipidemia, smoking) are potential confounders. In multivariable models, insulin use and hypertension were balanced across CRP quartiles in our cohort, suggesting they did not drive the association. Similar to our study, prior analyses have adjusted for these factors (age, sex, HbA1c, blood pressure, lipid levels) when testing CRP–DR links.^15^ In summary, subgroup trends indicate that while hs-CRP correlates with several DR risk factors, its independent association with DR severity is not explained by any single demographic or clinical factor. There are several ways in which this study advances the existing literature in this area. First, we employed a multivariable logistic regression model to adjust for important clinical and metabolic confounding factors (age, sex, duration of diabetes, HbA1c, BMI, hypertension, and lipid levels), which were inconsistently adjusted for in previous studies. The continued presence of a significant association between hs-CRP and DR after adjustment provides increased confidence for an independent association between systemic inflammation and retinal disease. Second, we studied a well-defined clinical cohort from a Middle Eastern population that is underrepresented in CRP-DR studies to increase the international applicability of the results. Third, the DR grading used in our study was performed by fellowship-trained retina specialists using an obligatory, standardized examination performed with 7-field photographs, increasing consistency and accuracy of diagnosis relative to earlier studies that relied upon self-report or less descriptive measures. When considered together, these methodological strengths serve to improve the validity and interpretation of our results and assist in resolving prior inconsistencies in the literature.

 There are several biologically plausible pathways by which CRP could be associated with the neurodegeneration and capillary dropout seen in retinopathy. Systemically, CRP is synthesized by hepatocytes (and adipocytes) in response to interleukin-6, IL-1β, and TNF-α.^16^ Once in the circulation, CRP can exert direct effects on endothelial cells and immune cells via Fcγ receptors (especially CD32) and complement activation. For example, CRP has been shown to directly upregulate endothelial adhesion molecules such as ICAM-1, VCAM-1, and E-selectin. This upregulation facilitates leukocyte adherence to the retinal microvascular endothelium, leading to leukostasis and capillary occlusion, hallmark features of early DR. CRP also stimulates secretion of chemokines like MCP-1,^19^ further attracting inflammatory cells into the retinal tissue. Thus, elevated CRP can actively promote the low-grade endothelial dysfunction and leukocyte-mediated capillary damage observed in DR. On a molecular signaling level, CRP binding to FcγR on retinal endothelial and glial cells triggers intracellular pathways. Qiu et al showed in transgenic rats that human CRP activates the NF-κB pathway via CD32 receptors.^21^ This leads to overproduction of pro-inflammatory cytokines (e.g. TNF-α, IL-1β) and chemokines in the retinal milieu. In their study, CRP-overexpressing diabetic rats had greatly increased retinal levels of TNF-α and ICAM-1 (and leukocyte adhesion) compared to controls, as well as enhanced oxidative stress (reactive oxygen species generation) in retinal cells. These changes mirror known DR mechanisms: elevated TNF-α and ROS are implicated in blood-retinal barrier breakdown and neurovascular injury. In fact, CRP has been shown to generate superoxide in vascular cells, impairing nitric oxide–mediated vasodilation.^19^ These pro-oxidant effects would exacerbate microvascular pathology. In sum, by amplifying NF-κB–driven inflammation and oxidative stress, CRP plausibly accelerates the neurodegeneration and capillary dropout seen in retinopathy. Antioxidant mechanisms, such as those involving selenoproteins and other endogenous regulators, may offer a therapeutic counterbalance to this inflammatory cascade.^22^

 CRP may also be associated with modulation of the angiogenic drive in ischemic retinopathy, potentially involving cytokine signaling, oxidative injury, and VEGF-driven angiogenesis. The study by Qiu et al found that CRP-overexpressing rats had markedly more retinal neovascularization in an oxygen-induced retinopathy model, implying a pro-angiogenic effect. Mechanistically, CRP can upregulate vascular endothelial growth factor (VEGF). We observed (and Qiu et al confirm) that overexpression of CRP led to higher retinal VEGF levels under ischemic conditions.^21^ This may occur via indirect ischemia and also direct signaling: in cultured cells, CRP has been shown to induce VEGF expression through activation of CD64 and the PI3K/Akt/HIF-1α pathway. Thus, CRP can enhance pathological retinal neovascularization, a key feature of proliferative DR. Moreover, CRP is found locally in the eye: studies have detected CRP in the vitreous fluid of PDR eyes at concentrations comparable to the serum,^16^ suggesting that systemic inflammation may translate to the intraocular compartment, where CRP could exert local effects on retinal endothelium and pericytes. Collectively, these mechanistic insights support CRP as an active participant in DR pathogenesis, affecting endothelial dysfunction, cytokine signaling, oxidative injury, and VEGF-driven angiogenesis. Similar oxidative stress mechanisms have also been implicated in other ocular diseases, such as cataract formation, where antioxidant therapies are being explored as potential interventions.^23^

 Some authors have proposed CRP as a non-invasive systemic biomarker of retinopathy. The meta-analysis by Song et al suggested that CRP may be used as a biomarker to determine the severity of DR.^17^ Indeed, in cross-sectional screening, elevated hs-CRP might flag patients who deserve more careful retinal evaluation or earlier intervention. Prospective data are suggestive but limited. In the Diabetes Control and Complications Trial (DCCT) cohort of type 1 diabetics, higher baseline hs-CRP predicted the development of clinically significant ME (a vision-threatening DR complication).^24^ This supports the idea that systemic inflammation markers could forecast retinal outcomes.^25^ However, CRP is a very non-specific marker of inflammation; it rises with infection, other vascular diseases, obesity, and metabolic syndrome. Thus, its positive predictive value for DR in isolation would be low. Moreover, as noted, published studies on CRP and DR have been inconsistent.^16^ In practice, then, hs-CRP might at best serve as one component of a multifactorial risk model (along with HbA1c, blood pressure, lipid profile, duration, etc).^11^ From a clinical perspective, hs-CRP is potentially a low-cost and readily available biomarker for addressing the risk of DR in individuals with T2DM, particularly in settings where imaging of the retina is impractical. Importantly, hs-CRP is not a substitute for fundus examination or imaging; however, elevated levels of hs-CRP may encourage clinicians to consider obtaining retinal images sooner and, for high-risk patients, more often. hs-CRP might also be used in a multivariable risk model with other established factors (HbA1c level, duration of diabetes, and blood pressure) to improve DR screening procedures. However, before clinical implications can be suggested for hs-CRP, important prospective studies are needed to assess whether hs-CRP improves prediction or provides additional value beyond currently available risk scores. In addition to CRP, other systemic or environmental stimuli that contribute to vascular inflammation may also play a role in DR pathogenesis. For example, endocrine-disrupting chemicals can cause mitochondrial dysfunction and exacerbate inflammatory damage in the microvascular complications of diabetes.^26^ These mechanisms should be considered in broader models of DR pathogenesis.

 Several limitations of our study must be acknowledged. First and foremost is the cross-sectional design, which limits causal inference. We can only demonstrate that higher hs-CRP coexists with more severe DR, not that CRP elevation precedes or causes retinopathy. Reverse causation is possible (e.g. retinal ischemia could induce systemic inflammation), and unmeasured confounders may influence both CRP and DR. Although we adjusted for major confounders (age, sex, HbA1c, BMI, blood pressure, medication use), residual confounding by factors like smoking, statin use, or unrecognized inflammatory conditions cannot be excluded. We excluded subjects with extremely high CRP ( > 10 mg/L) to minimize acute infection bias, but low-grade infections or other chronic diseases might still elevate CRP. Second, single measurements of hs-CRP may misclassify chronic inflammation; ideally, serial measurements would confirm persistent elevation. Third, our sample may limit generalizability. This was a single-center study in a specific ethnic population; CRP levels and DR risk could differ in other demographic groups. Fourth, we did not assess other inflammatory markers (e.g. IL-6, TNF-α, adhesion molecules) that might provide a more complete picture of vascular inflammation. Finally, we did not evaluate retinal imaging biomarkers (such as OCT angiography) that could correlate with CRP. These limitations are similar to those noted in other observational studies of DR.^17,27,28^ Also, we suggest categorizing the type of medications used for T2DM (e.g. statins, etc) for determining the role of different classifications of drugs in the progression of DR. Taken together, our results should be interpreted as evidence of association rather than proof of causation, and should prompt further investigation.

 To build on these findings, future research should adopt longitudinal and interventional approaches. A prospective cohort study measuring baseline hs-CRP (and other cytokines) in diabetic patients and following them for incident DR and progression would clarify temporal relationships. The DCCT experience suggests that hs-CRP can predict specific DR outcomes in type 1 diabetes^24^; similar studies in T2DM are needed. Genetic analyses could also help test causality. For example, a polymorphism in the CRP gene (rs2808629) was recently linked to higher DR risk in a Chinese cohort, implying a genetic predisposition mediated by CRP.^15^ Mendelian randomization studies using CRP-related single-nucleotide polymorphisms could probe whether lifelong higher CRP causally increases DR risk. Interventional trials would provide the most definitive evidence. Clinical trials of anti-inflammatory or CRP-lowering therapies (for instance, IL-6 receptor antagonists, statins, or novel CRP inhibitors) with DR endpoints would test whether dampening systemic inflammation can slow retinopathy. Given CRP’s biological involvement, targeting CRP–CD32/NF-κB signaling in animal models has shown promise^21^; translating such strategies to humans (e.g. evaluating approved anti-inflammatory agents) is an intriguing direction. Finally, integrated biomarker approaches should be explored: CRP might be combined with other markers (such as interleukins or endothelial function markers) to form a risk score. Imaging studies correlating CRP with retinal microvascular changes (e.g. on OCT-angiography) may also yield new insights. Quercetin-based therapies, shown to reduce ferroptosis and oxidative stress in diabetic encephalopathy, may also hold therapeutic potential in DR due to shared inflammatory and redox mechanisms.^29^

## Conclusion

 Our findings reinforce that systemic inflammation, as reflected by hs-CRP, is linked with more severe DR. This adds to a growing body of evidence that chronic inflammation contributes to the microvascular complications of diabetes. While causality remains to be established, considering inflammatory pathways alongside traditional risk factors may improve our understanding of DR pathogenesis. Future longitudinal and mechanistic studies, including prospective cohorts, genetic analyses, and interventional trials, are warranted to clarify whether hs-CRP can serve as a useful biomarker and therapeutic target in diabetic eye disease.
